# Rejuvenation: Turning Back Time by Enhancing CISD2

**DOI:** 10.3390/ijms232214014

**Published:** 2022-11-13

**Authors:** Chi-Hsiao Yeh, Zhao-Qing Shen, Ching-Cheng Lin, Chung-Kuang Lu, Ting-Fen Tsai

**Affiliations:** 1 Department of Thoracic and Cardiovascular Surgery, Chang Gung Memorial Hospital, Linkou 333, Taiwan; 2 College of Medicine, Chang Gung University, Taoyuan 333, Taiwan; 3 Department of Life Sciences and Institute of Genome Sciences, National Yang Ming Chiao Tung University, Taipei 112, Taiwan; 4 National Research Institute of Chinese Medicine, Ministry of Health and Welfare, Taipei 112, Taiwan; 5 Institute of Molecular and Genomic Medicine, National Health Research Institutes, Miaoli 350, Taiwan; 6 Center for Healthy Longevity and Aging Sciences, National Yang Ming Chiao Tung University, Taipei 112, Taiwan

**Keywords:** CISD2, aging, rejuvenation, longevity, mitochondria, calcium homeostasis, hesperetin

## Abstract

The aging human population with age-associated diseases has become a problem worldwide. By 2050, the global population of those who are aged 65 years and older will have tripled. In this context, delaying age-associated diseases and increasing the healthy lifespan of the aged population has become an important issue for geriatric medicine. CDGSH iron-sulfur domain 2 (CISD2), the causative gene for Wolfram syndrome 2 (WFS2; MIM 604928), plays a pivotal role in mediating lifespan and healthspan by maintaining mitochondrial function, endoplasmic reticulum integrity, intracellular Ca^2+^ homeostasis, and redox status. Here, we summarize the most up-to-date publications on CISD2 and discuss the crucial role that this gene plays in aging and age-associated diseases. This review mainly focuses on the following topics: (1) CISD2 is one of the few pro-longevity genes identified in mammals. Genetic evidence from loss-of-function (knockout mice) and gain-of-function (transgenic mice) studies have demonstrated that CISD2 is essential to lifespan control. (2) CISD2 alleviates age-associated disorders. A higher level of CISD2 during natural aging, when achieved by transgenic overexpression, improves Alzheimer’s disease, ameliorates non-alcoholic fatty liver disease and steatohepatitis, and maintains corneal epithelial homeostasis. (3) CISD2, the expression of which otherwise decreases during natural aging, can be pharmaceutically activated at a late-life stage of aged mice. As a proof-of-concept, we have provided evidence that hesperetin is a promising CISD2 activator that is able to enhance CISD2 expression, thus slowing down aging and promoting longevity. (4) The anti-aging effect of hesperetin is mainly dependent on CISD2 because transcriptomic analysis of the skeletal muscle reveals that most of the differentially expressed genes linked to hesperetin are regulated by hesperetin in a CISD2-dependent manner. Furthermore, three major metabolic pathways that are affected by hesperetin have been identified in skeletal muscle, namely lipid metabolism, protein homeostasis, and nitrogen and amino acid metabolism. This review highlights the urgent need for CISD2-based pharmaceutical development to be used as a potential therapeutic strategy for aging and age-associated diseases.

## 1. Introduction

### 1.1. CISD2 Is One of a Limited Number of Pro-Longevity Genes in Mammals

According to an estimate by the United Nations, the aging population is globally increasing both in numbers and as a proportion of the total world population [1]. People aged 65 years or above in the global population are projected to rise from 10% in 2022 to 16% in 2050. The lifelong accumulation of molecular and cellular damage during aging results in progressive co-morbidities that affect multiple organ systems. This co-occurrence of multiple age-associated diseases increases mortality and morbidity within aged populations [2].

Genome-wide association studies have revealed that there is a lifelong survival advantage among members of long-lived families and this has been attributed to a lower risk of coronary artery disease, cancer, and type 2 diabetes [3,4]. Two hypotheses have been proposed to explain the above observations. The first is the presence of alleles protecting against these diseases that contribute to population mortality, and the other is the absence of alleles promoting such diseases. However, many studies have shown that a decreased prevalence of cardiovascular disease [5], hypertension, and type 2 diabetes exists in the offspring of nonagenarian siblings and centenarians [4], which suggests that the absence of these disease loci does not explain the lower morbidity among these offspring. A lower cancer mortality rate has been observed in the offspring of centenarians, despite the same cancer prevalence when compared with controls [6]. Exploring the potential mechanisms of health-maintaining genetic variants among individuals, who seem to be safeguarded from diseases, remains critical [7,8]. Therefore, protective alleles may be present in long-lived individuals and these are likely to be able to delay the onset of or decrease the severity of age-associated diseases [9]. Currently, most (up to 90%) of the therapeutic agents entering drug-development pipelines fail. Accordingly, developing new and effective medicines to treat age-associated diseases by exploiting the protective alleles of health-maintaining genes could provide an alternative means of extending healthspan [8,10].

Currently, only eight genes (*BUB1B*, *CISD2*, *KLOTHO*, *PAWR*, *PPARG*, *PTEN*, *SIRT1*, and *SIRT6*) are listed as pro-longevity genes by the Human Aging Genomic Resources, which means that they have been experimentally demonstrated to mediate lifespan in mammals. Genetic knockout of these pro-longevity genes has been found to result in a shortened lifespan in mice; conversely, transgenic overexpression of pro-longevity genes have effectively extended lifespan in mice [11]. Interestingly, when using calculations based on the Gompertz mortality rate model, which is one of the most well-known mortality models, two genes, namely *CISD2* and *hMTH1*, stand out and are suggested to slow down aging [12,13,14]. Indeed, our mouse studies have revealed that a higher level of CISD2 expression is able to slow down natural aging and promote longevity, as well as delay the onset of and reduction in severity of age-associated diseases [14,15,16,17,18].

### 1.2. Functions of CISD2

Currently, the CDGSH iron-sulfur domain (CISD) protein family is known to contain three genes that are present in mammals, namely CISD1 (a.k.a., C10orf70, MDS029, ZCD1, and mitoNEET), CISD2 (a.k.a., ERIS, ZCD2, NAF-1, WFS2, and Miner1), and CISD3 (a.k.a., MiNT and Miner2). All members of the CISD protein family contain the CDGSH (Cys-Asp-Gly-Ser-His) 2Fe-2S domain, which is highly conserved in mammals. Both the CISD1 and CISD2 proteins contain a transmembrane (TM) domain and a single CDGSH iron-sulfur domain. On the other hand, CISD3 has two CDGSH iron-sulfur domains and lacks the TM domain. The subcellular localization of the CISD1 and CISD3 proteins are the mitochondrial outer membrane (MOM) and matrix, respectively [19]. The subcellular localization of the CISD2 protein is quite diverse, including the MOM, endoplasmic reticulum (ER), and mitochondrial-associated ER membranes (MAM) [20]. The unique features of these subcellular locations means that the CISD2 protein is able to mediate functions that are distinct from those of its paralogs, these include MAM integrity and ER Ca^2+^ homeostasis. In mouse studies, CISD1 deficiency appears to result in a number of distinct phenotypes, including promoting Parkinson’s disease-like neurodegeneration and accelerating age-related heart failure [21,22,23]. Until now, the function(s) of CISD3 protein have remained largely unexplored. On the other hand, CISD2 deficiency has been shown to cause a variety of age-related phenotypes in multiple organs, which lead to premature aging in mice [20].

#### 1.2.1. CISD2 Is the Disease Gene of WFS2 in Humans

A homozygous mutation of the CISD2 gene was identified in three related families of patients with Wolfram syndrome 2 (WFS2; MIM 604928) [24]. These patients developed early onset diabetes mellitus in their first or second decade of age, experienced vision loss due to optic atrophy, and had high-frequency sensorineural hearing loss; these phenotypes affect almost all of the patients. Other abnormalities among these individuals included urinary tract dilatation (8/16, 50%), upper gastrointestinal ulceration (10/11, 91%), and defective platelet aggregation with a tendency to bleed (11/14, 79%). However, none of the patients developed diabetes insipidus [25].

#### 1.2.2. CISD2 Maintains Mitochondrial and MAM Integrity

Our previous studies have shown that CISD2 is a nucleus-encoded mitochondrial protein that is directed to localize on the mitochondrial outer membrane (MOM) by its N-terminal 58 amino acids [26,27]. A portion of expressed CISD2 has been also identified on the endoplasmic reticulum (ER) and mitochondrial-associated ER membranes (MAMs) [26,28]. The location of CISD2 proteins on MOMs and MAMs is closely related to their biological functions. Loss of CISD2 results in the breakdown of the MOM before the destruction of the inner cristae of the mitochondria. These abnormalities affecting mitochondrial structures are accompanied by an impairment of mitochondrial function in skeletal muscle, adipose tissue, the diaphragm, heart, and liver [17,18,29,30]. Subsequently, mitochondrial degeneration appears to induce autophagy, and this causes an elevated level of oxidative stress, which can result in cell death [18,27]. In addition, CISD2 deficiency seems to lead to defective inter-organellar communication, including the mitochondria–lysosomal and mitochondria–ER crosstalk in the heart [28]. This impaired mitochondria–lysosomal crosstalk, in turn, compromises lysosomal enzymatic activity and affects various autophagic pathways, thereby resulting in an imbalance in lipid metabolism and dysregulation of cellular proteostasis [28].

#### 1.2.3. CISD2 Regulates Intracellular Ca^2+^ Homeostasis

The dynamic characteristics of MAMs, which are present between the mitochondria and ER, provide an efficient way for Ca^2+^ trafficking between these two organelles; this generates a higher Ca^2+^ microenvironment close to the MAMs compared with the surrounding cytoplasm [31]. A decrease in the level of CISD2 expression during aging results in a wider distance between the ER and mitochondria and thus a higher cytosolic Ca^2+^ concentration [18,26,29]. Our previous studies have revealed that CISD2 is involved in the maintenance of intracellular Ca^2+^ homeostasis in several different cell types, including hepatocytes [29] and cardiomyocytes [18]. The sarco/endoplasmic reticulum Ca^2+^-ATPase (SERCA) is a Ca^2+^ channel containing a P-type ATPase domain; the major function of SERCA is to transport Ca^2+^ from the cytosol into the ER/SR. We have provided evidence showing that CISD2 interacts with SERCA2 to protect SERCA2 from irreversible oxidative modification that jeopardizes SERCA2’s enzymatic activity. Thus, CISD2 appears to regulate intracellular Ca^2+^ homeostasis via the modulation of the redox status of the SERCA2 protein, thus maintaining the enzymatic activity of SERCA2 [18,20,29,32].

Another CISD2 interacting protein is GIMAP5 (GTPase, IMAP family member 5), which is located on the MOM and MAM and is also involved in intracellular Ca^2+^ regulation [30]. GIMAP5 mediates the Ca^2+^ uptake capacity of the mitochondria. Loss of either CISD2 or GIMAP5 results in a decreased level of mitochondrial Ca^2+^ uptake and an elevated level of cytosolic Ca^2+^. Loss of both CISD2 and GIMAP5 at the same time causes a more severe defect that impairs intracellular Ca^2+^ homeostasis. In addition, a previous study has shown that BCL2 is one of the proteins that interact with CISD2. It seems that the BCL2–CISD2 complex physically associates with IP3R (inositol 1,4,5-trisphosphate receptor) to modulate the Ca^2+^ channel activity of IP3R [32,33,34]. Thus, CISD2 seems to widely participate in the regulation of intracellular Ca^2+^ homeostasis via its ability to interact with multiple Ca^2+^ channel proteins.

#### 1.2.4. CISD2 Modulates Redox Status

CISD2 was initially reported to be localized to the ER. Loss of CISD2 induced ER stress, which was identified by both transmission electron microscopy (TEM) and the presence of elevated levels of phosphorylated eIF2α (eukaryotic translation initiation factor 2, subunit 1 alpha) [17,29]. The 2Fe-2S cluster of the CISD protein participates in electron transfer reactions [35]. Mitochondrial complex I and III, especially complex I, are deemed to be the most important source of cellular ROS production [36]. A 30% decrease in the electron transport activity of mitochondrial complexes I–III, complexes II–III, and complex IV has been attributed to CISD2 deficiency [26]. Thus, a deficiency in CISD2 or a decrease in CISD2 levels leads to a functional reduction in mitochondrial oxidative phosphorylation, activation of ER stress, and dysregulation of intracellular Ca^2+^. These collectively contribute to enhanced oxidative stress and elevated ROS levels, which then result in dyshomeostasis of intracellular redox within adipose tissue [30], the liver [29,37], skeletal muscle [17], and the heart [18].

## 2. CISD2 Mediates Lifespan and Healthspan

An age-dependent decrease in CISD2 expression during the natural aging of mice has been reported in a range of tissues, including the brain, spinal cord, skeletal muscle, heart, and skin [14,17,18,27,38]. However, the rate of CISD2 downregulation varies from tissue to tissue [20].

### 2.1. CISD2 Is One of a Limited Number of Pro-Longevity Genes in Mammals

CISD2 deficiency is associated with many hallmarks of aging, including mitochondrial dysfunction accompanied by autophagy and cell death, disorganized proteostasis, deregulated nutrient sensing with metabolism disturbances, stem cell exhaustion, and alterations in intercellular communication [39]. The earliest manifestation of mitochondrial dysfunction appears in the organs with the highest energy demand, namely the heart, skeletal muscle, and nervous system. Furthermore, CISD2 knockout (CISD2 KO) mice display a panel of phenotypic features that are indicative of premature aging; these include a shortened lifespan accompanied by multiple age-associated organ dysfunctions (cardiac electromechanical dysfunction, sarcopenia, and degeneration of the nervous system), as well as dysregulation of whole body energy metabolism. Notably, many of these manifestations are consistent with the symptoms present in WFS2 patients. Moreover, in CISD2 transgenic (CISD2 TG) mice, our previous studies revealed that a persistently high level of CISD2 extended their median and maximum lifespan without any apparent deleterious side effects [14]. A persistently high level of CISD2 expression ameliorated age-associated degeneration of the skin, skeletal muscles, neurons, and cardiac performance during old age [14,17,18,40].

### 2.2. CISD2 in Cardiac Aging

**Figure 1:**
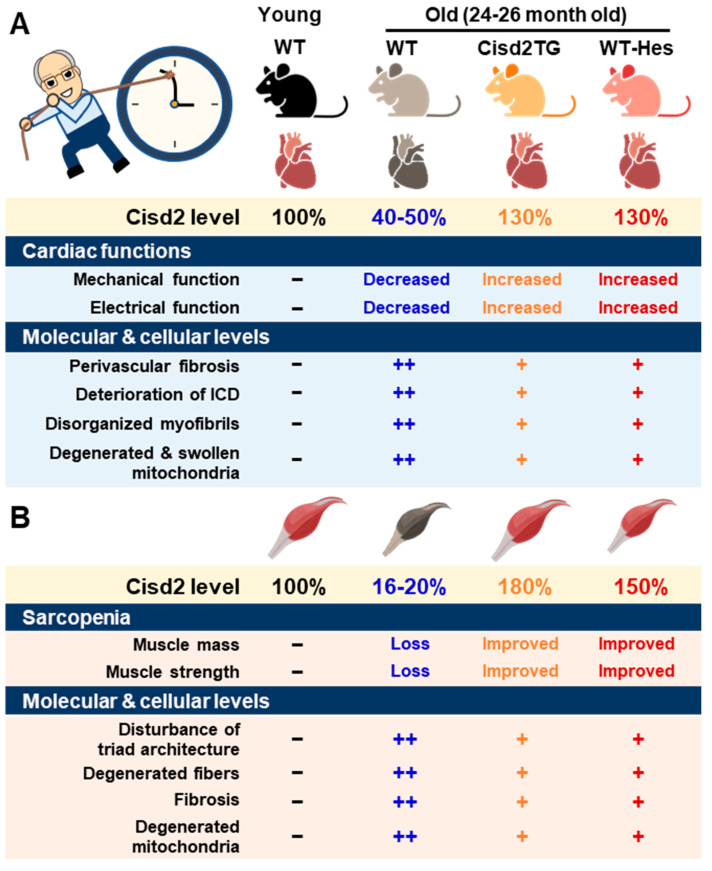
CISD2 attenuates heart and skeletal muscle aging. (**A**) An age-dependent decrease in CISD2 correlates with cardiac dysfunctions and molecular pathological alterations. Whole-body CISD2 overexpression (CISD2 TG) delays age-associated cardiac damage. In addition, the CISD2 activator, hesperetin (Hes), reverses age-associated cardiac damage via the activation of CISD2. (**B**) Age-dependent CISD2 reduction leads to sarcopenia and pathological alterations. Maintaining a high level of CISD2 by transgenic overexpression, or by hesperetin treatment, is able to protect skeletal muscle from age-related pathological and molecular damage. The figure was created with BioRender.com.

A major risk factor for cardiovascular disease is aging. Thus, it is critical to preserve cardiac functioning during aging [41]. One of our previous studies showed that CISD2 plays an essential role in maintaining normal cardiac structure and function [18]. The level of cardiac CISD2 present in naturally aged mice is significantly reduced by approximately 50% compared with that found in 3-month old (3-mo) young mice (Figure 1A). Loss of CISD2 in CISD2 KO mice resulted in an aged phenotype of the heart, including perivascular fibrosis, deterioration of the intercalated discs (ICDs), disorganized myofibrils, and degenerated and swollen mitochondria. Subsequently, these changes brought about myocardial degeneration and impairment of the electromechanical performance of CISD2 KO mice at a young age (3-mo). Maintenance of a high level of CISD2 protein in the heart is crucial to preserving the integrity of the ICDs, which allows for synchronous contraction and relaxation of cardiomyocytes as a single functional organ. ICD degeneration, including lateralization of gap junctions, maldistribution of desmosomes, and a breakdown of the fascia adherens, appears in CISD2 KO mice at a young age, due to the lack of CISD2, and in naturally aged mice, due to the decrease in CISD2 with aging. ICD degeneration leads to electromechanical impairment. Loss of CISD2 also results in derangement of mitochondrial ultrastructure and the abnormal functioning of oxidative phosphorylation. We have also shown that, in the heart, CISD2 directly interacts with SERCA2a to modulate its enzymatic activity via oxidative modifications of SERCA2a. Oxidative modification of SERCA2a resulted in a reduced Ca^2+^ uptake of ER/SR, which was accompanied by an elevated basal level of cytosolic Ca^2+^. Consequently, the elevated level of cytosolic Ca^2+^ led to mitochondrial Ca^2+^ overload and compromised mitochondrial functioning.

On the other hand, a persistently high level of CISD2 during old age, which was achieved by a transgenic approach in the CISD2 TG mice, appears to preserve cardiac functions during aging [18,42]. Intriguingly, cardiac-specific induction of CISD2 overexpression at 18 mos of age appeared to retard cardiac aging and reverse age-associated cardiac dysfunction to a measurable extent in mice. Enhanced expression of CISD2 at a late-life stage is thus able to attenuate the age-associated decline in mitochondrial membrane potentials and respiratory function, as well as minimize ROS production, thereby rejuvenating an aging heart [42].

### 2.3. CISD2 in Muscle Aging

One of the most prominent features during aging is the loss of skeletal muscle mass and strength/function, which is referred to as sarcopenia [43]. The proteomic signature of sarcopenia reveals that mitochondrial dysfunction and alterations in intracellular Ca^2+^ homeostasis are significantly associated with sarcopenia [44,45]. The preferential loss of muscle during aging involves the glycolytic fast-twitch fibers of the gastrocnemius muscle, in particular [46,47].

There was a significant downregulation of CISD2 protein in skeletal muscle, specifically the gastrocnemius, of WT mice at middle age (at 13 mo; only ~30% of CISD2 protein remained) and old age (at 24 mo; only 16 to 20% of CISD2 protein remained), which suggests that CISD2 may play a vital role in skeletal muscle aging (Figure 1B) [17,20]. Notably, mice carrying the muscle-specific knockout of CISD2 (CISD2 mKO) at a young age (3 mo) showed similar phenotypic characteristics to those observed in naturally aged mice; this includes degeneration of skeletal muscles, which is accompanied by impairment of proteostasis and destruction of mitochondria and the ER/SR. In these CISD2 mKO mice, CISD2 deficiency induced ER stress and unfolded protein response (UPR) signaling in the muscle at a young age (3 mo). In addition, CISD2 deficiency led to alterations in the antioxidant defense pathways of both young CISD2 mKO at 3 mo and naturally aged WT mice at 24–26 mo. Subsequently, the resulting elevated ROS levels appeared to increase the oxidative modifications of SERCA1, thereby damaging enzymatic activity of this enzyme, which brought about an impairment of Ca^2+^ re-uptake into the ER/SR. This reduction in the Ca^2+^ level of the ER/SR appeared to compromise the functioning of many chaperones, which are proteins that assist others to fold properly; this folding is Ca^2+^-dependent. The end result was that these abnormalities formed a vicious cycle that was exacerbated during aging.

Intriguingly, in CISD2 TG mice, which are a long-lived mouse model, a persistently high level of CISD2 expression was able to protect skeletal muscles from age-dependent mass loss and functional decline [14]. TEM examination further revealed that CISD2 appears to ameliorate the age-associated ultrastructural abnormalities normally present in the skeletal muscles, namely a disturbance of triad structure, disorganization of fibers, and degeneration of mitochondria [14].

### 2.4. CISD2 in Liver Aging

The liver is one of the most important metabolic organs for maintaining whole body health and plays a pivotal role in several biological processes, such as lipid metabolism, glucose homeostasis, xenobiotic and drug detoxification, as well as in the biosynthesis and secretion of plasma proteins. Accordingly, age-associated liver dysfunction not only affects the liver per se, but also contributes to the development of many other age-related diseases, such as various cardiovascular diseases, metabolic syndrome, diabetes, and a number of cancers. During liver aging, multiple age-related alterations, including pathological changes, functional decline, and metabolic dysregulation have been found [48]. Regarding the hallmarks of aging in the liver, there are several remarkable changes; these include a loss of proteostasis, the induction of ER stress, presence of dysfunctional mitochondria, increase in oxidative stress, and presence of cellular senescence. In particular, the mitochondria appear to play a crucial role in the aging of the liver [48,49].

Previously, we demonstrated that CISD2 maintained cellular homeostasis via the modulation of mitochondrial function, ER integrity, redox status, and intracellular Ca^2+^ homeostasis [20]. Intriguingly, in the aging liver, the level of CISD2 protein has been found to decrease to about 50% in old mice at 26 mo compared with young mice at 3 mo [50]. This result suggests that an age-associated decline in CISD2 expression seems to be involved in the liver aging process. Importantly, in Cisd2 TG mice at 26 mo, a persistently high level of CISD2 was shown to attenuate age-related liver pathology, reduce oxidative stress, and preserve a youthful pattern of gene expression, as revealed by transcriptomic and proteomic analyses. Such an effect thereby maintains normal metabolic function within the liver during aging [40,50]. Mechanistically, CISD2 seems to suppress the age-related dysregulation of various transcription mediators, such as Nrf2, IL-6, and Hnf4a; this, in turn, may help to preserve the transcriptional network, thereby resulting in a younger profile of gene expression in the liver. Interestingly, CISD2 appears to function in a cell autonomous manner when protecting hepatocytes from age-associated damage. In the AML12-CISD2 KO hepatocyte cell line, CISD2 deficiency resulted in lipid accumulation, mitochondrial dysfunction, and increased oxidative stress; additionally, CISD2 deficiency also led to dysregulation of the downstream target genes of Nrf2 and Hnf4a. On the other hand, in the AML12-CISD2 RE hepatocyte cell line, in which CISD2 was re-expressed in the CISD2 KO hepatocytes, all of the cellular phenotypes disappeared [50]. Furthermore, our previous studies revealed that CISD2 heterozygous knockout (CISD2^+/−^) mice were more susceptible to developing non-alcoholic fatty liver disease (NAFLD) and non-alcoholic steatohepatitis (NASH); these liver phenotypes are exacerbated during aging. Indeed, CISD2 haploinsufficiency disrupts calcium homeostasis, induces ER stress, and promotes hepatocellular carcinoma (HCC). In contrast, CISD2 overexpression prevents HCC development and protects against HBx-mediated liver damage and lipotoxicity [29].

Taken together, genetic evidence reveals that downregulation of CISD2 accelerates liver aging and promotes the development of age-related liver diseases, including NAFLD, NASH, and HCC. Conversely, a high level of CISD2 protects the liver from oxidative stress, preserves mitochondrial functioning, and attenuates the age-related loss of proteostasis, as well as maintains a youthful pattern of gene expression [40,50].

## 3. CISD2 Alleviates a Range of Age-Associated Disorders

Aging is the main risk factor for many diseases, for example, Alzheimer’s disease (AD), NAFLD, NASH, as well as various eye diseases. Thus, we have explored the beneficial effects and molecular mechanisms by which CISD2 has an impact on certain age-associated diseases that can be studied in mice.

### 3.1. CISD2 Improves the Outcome of Alzheimer’s Disease in Mice

Alzheimer’s disease, or AD, which is characterized by loss of memory and deterioration in cognitive function, constitutes about two-thirds of cases of dementia among older adults [51]. In naturally aged WT mice at 24 mo, the degeneration of non-myelinated and myelinated axons and disintegration of the myelin sheath can be easily detected in the sciatic nerve, which belongs to the peripheral nervous system, and in the optic nerve, which is an extension of the central nervous system. However, in the CISD2 TG mice at 24 mo, a persistently high level of CISD2 was able to protect the sciatic and optic nerves from age-associated degeneration [14]. Accordingly, there has been a lot of interest in whether an increased level of CISD2 can protect the brain from Aβ-mediated toxicity, reducing neuronal damage in the hippocampus, thereby improving the AD phenotype by attenuating AD pathogenesis within the brain. Remarkably, in an AD mouse model, namely APP/PS1 double transgenic mice, our studies revealed that CISD2 upregulation in AD mice promoted their survival and protected against neuronal loss. A dose-dependent therapeutic effect of CISD2 exists and this is able to modulate the severity of the AD phenotype [15]. Notably, CISD2 deficiency accelerates Aβ-mediated pathogenesis in the AD brain. Conversely, a transgenic increase in the level of CISD2 protects mitochondria and attenuates the loss of neuronal progenitor cells. Intriguingly, CISD2 upregulation shifts the expression patterns of the dysregulated genes found in AD mice toward that of WT mice [15]. Altogether, these genetic studies provide evidence for and indicate that CISD2 overexpression slows down the progression of AD in mice and that CISD2-based therapies are likely to hold great promise as a potential therapeutic strategy for treating AD.

### 3.2. CISD2 Ameliorates Fatty Liver Disease

The overall global prevalence of NAFLD diagnosed by imaging is around 25.24% [52]. The global prevalence of NASH, which is a more serious form of fatty liver disease, ranges between 1.5 and 6.45% in the general population [52]. NAFLD is the most common liver disease in society, with the highest prevalence being in those over 60 years old [53]. NASH is characterized by hepatocytic ballooning, hepatocyte death, inflammatory cell infiltration, and fibrosis. In the United States, NAFLD is the third most common cause of HCC [54]. Interestingly, the severity of Western diet (WD)-induced NAFLD is negatively correlated with the expression levels of CISD2 in the liver [37]. Specifically, the development and progression of fatty liver toward NASH are accelerated by CISD2 haploinsufficiency, which leads to abnormalities in the ER and disturbances in intracellular Ca^2+^ homeostasis within hepatocytes. Conversely, a high level of CISD2 is able to protect the liver from oxidative stress, reduce the occurrence of mitochondrial DNA deletions, and attenuate the pathogenesis of NAFLD and NASH [29]. Altogether, our mouse studies show that CISD2 plays a pivotal role in protecting the liver from WD-induced NASH, and that CISD2 is a promising molecular target for the development of therapeutic agents for the treatment of WD-induced fatty liver diseases [37,55].

### 3.3. CISD2 Maintains Corneal Epithelial Homeostasis

Aging of the ocular surface and corneal tissues is a major cause of eye disease, including the highly prevalent dry eye disease and corneal epithelial defects. In the United States, 14.4% of the population older than 50 years have dry eye disease [56]. Our study revealed that CISD2 was crucial to the maintenance of corneal epithelial integrity and repair. In mice, CISD2 deficiency caused their ocular surface to become vulnerable to injury and jeopardized the regeneration of corneal epithelial cells; this led to a vicious cycle of chronic injury and persistent repair. Consequently, this resulted in exhaustion of the limbal progenitor cells, thereby compromising the wound healing of the cornea [57]. Notably, CISD2 is downregulated in the corneal epithelial cells of patients with corneal epithelial diseases. However, CISD2 continues to be expressed at a significant level in the epithelial layer of patients with ocular diseases that only affect the corneal endothelial cells. In a human corneal epithelial cell line, namely HCEC, loss of CISD2 impaired mitochondrial function, disturbed intracellular Ca^2+^ homeostasis, and increased oxidative stress, thereby retarding corneal regeneration. Our study also revealed that cyclosporine A, which is an inhibitor of Ca^2+^-calcineurin-dependent signaling, facilitates wound healing of the corneal epithelial layer in CISD2 KO mice. Moreover, cyclosporine A treatment is also able to restore the corneal epithelium in patients with dry eye disease, which affects the ocular surface via corneal epithelial erosion [57]. These findings reveal that CISD2 plays an essential role in the cornea via maintaining corneal epithelial homeostasis and that CISD2 is a potential target for developing therapeutics to treat corneal epithelial diseases.

## 4. Regimens or Treatments That Enhance CISD2 Gene Expression

### 4.1. Weight Loss Surgery Restores CISD2 Levels in Obese Subjects

#### 4.1.1. Obesity Is One of the Major Risk Factors That Accelerate Age-Associated Diseases

Previous studies have revealed that obesity increases the risk of numerous age-associated diseases, such as cardiovascular disease, hypertension, AD, various cancers, and type 2 diabetes mellitus. Obesity and aging have several similar features including impairment of mitochondrial function, redox imbalance, accumulation of intracellular macromolecules and increased ER stress, as well as increased systemic inflammation, all of which result in an accelerated aging process and exacerbation of age-associated diseases [58]. Therefore, weight loss by lifestyle or medical interventions may be able to attenuate aging and prevent age-associated disorders.

#### 4.1.2. CISD2 Is Downregulated in Subjects with Morbid Obesity, Whereas Weight Loss Surgery Restores CISD2 Expression in Obese Humans

Using quantitative mass spectrometry analysis, Campbell et al. studied the alterations that occur in the proteomic profiles of the skeletal muscles of mid-aged obese female subjects (45.1 ± 3.6 years old; BMI > 40 kg/m^2^) before and 3 months after Roux-en-Y gastric bypass (RYGB) surgery. They found that 395 proteins were significantly different in skeletal muscle (135 upregulated and 260 downregulated) between the pre-surgery obese and age-matched lean control female subjects (48.5 ± 4.7 years old; BMI < 25 kg/m^2^). The level of CISD2 protein was found to be significantly decreased by 32.3% (*p* = 0.026) in the pre-surgery obese subjects. Intriguingly, 3 months after RYGB surgery (BMI = 35.3 ± 1.8 kg/m^2^), CISD2 protein in post-operative obese patients was increased to a level comparable to that of the lean control subjects [59]. This result indicates that being overweight, especially being morbidly obese, appears to downregulate CISD2 expression, whereas reducing body weight restores the expression level of CISD2 in humans (Table 1 (C)).

### 4.2. Exercise Attenuates Aging and Enhances CISD2 Gene Expression

Numerous studies have shown that lifestyle interventions, including diet restriction and exercise, delay aging and prevent age-associated diseases [60,61,62,63,64]. Exercise has several anti-aging effects and seems to do this by modulating the hallmarks of aging and reversing age-associated metabolic decline [61,62]. Interestingly, one of our studies has revealed that the expression levels of CISD2 in mouse skeletal muscle can be increased by exercise.

#### 4.2.1. Exercise Is a Promising Lifestyle Intervention That Is Able to Slow Down Aging

Previous studies have shown that exercise is a well-established anti-aging strategy that promotes longevity and healthspan, as well as helping to hold age-associated diseases at bay. The beneficial effects of physical exercise on the aging process of organisms have often been identified, and such benefits include improved executive functioning and memory, improved cardiopulmonary functioning, enhanced metabolism, better glucose homeostasis, and alleviation of skeletal muscle degeneration [61]. The benefits of exercise are highly associated with improvements in hallmarks of aging, including less genomic instability, lower telomere attrition, fewer epigenetic alterations, a reduced decline in proteostasis, reduced dysregulation of nutrient sensing, reduced mitochondrial dysfunction, less cellular senescence, less stem cell exhaustion, and improved intercellular communication [65]. In skeletal muscle, exercise ameliorates age-associated skeletal muscle dysfunction via modulation of the following aspects of mitochondrial biology: increasing the mitochondrial unfolded protein response, enhancing mitochondrial dynamics and biogenesis, promoting mitophagy, and maintaining integrity of MAMs [62]. Thus, mitochondria appear to play an important role in exercise-mediated healthy longevity.

#### 4.2.2. Exercise Enhances CISD2 Expression and Attenuates Skeletal Muscle Aging

One of our studies indicated that treadmill exercise for 8 weeks was able to enhance CISD2 gene expression at the transcriptional level. Moreover, we found that long-term exercise in mice appeared to improve various age-associated phenotypes, namely increasing the percentage of lean mass, reducing the percentage of fat mass, enhancing the regeneration of skeletal muscle after injury, and restoring a younger transcriptomic pattern within the skeletal muscle [66]. Furthermore, a previous study in mice showed that voluntary exercise training for 4 weeks using a running wheel increased the expression levels of CISD2 in skeletal muscle and epididymal white adipose tissue [67]. These findings suggest that the beneficial effects of exercise are likely to be associated with an enhanced level of CISD2 expression (Table 1 (B)).

### 4.3. Natural Compounds That Can Upregulate CISD2 Expression

Several naturally derived compounds have been identified to increase CISD2 gene expression, including hesperetin, curcumin, wild bitter melon (WBM) extract, α-eleostearic acid, egg shell membrane (ESM) powder, and hydrated ESM (Table 1 (B)). In addition to hesperetin, which will be discussed in Section 5, the other compounds mentioned above are briefly described below.

#### 4.3.1. Curcumin Increases CISD2 Expression

**Figure 2:**
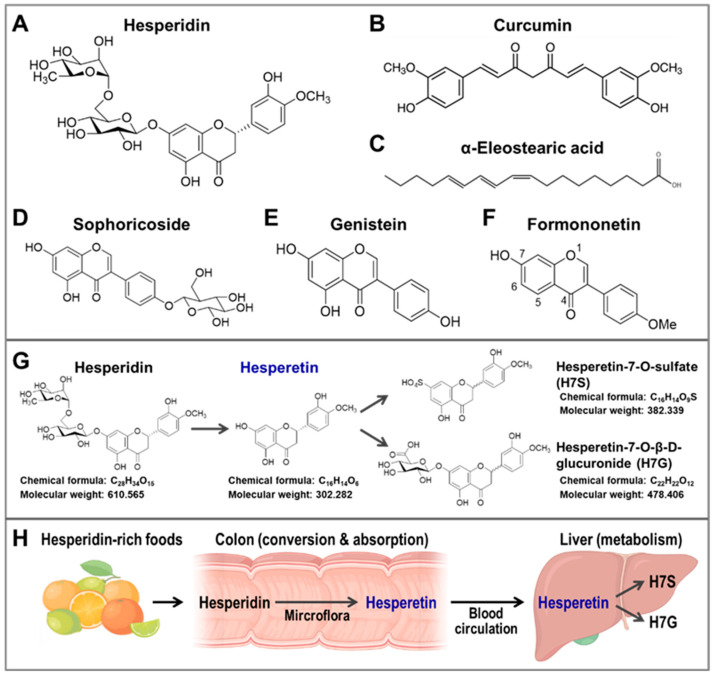
The chemical structures of the plant bio-activators of CISD2. The metabolism and bioavailability of hesperidin and its occurrence in foods. (**A**–**F**) The chemical structures of (**A**) hesperidin, (**B**) curcumin, (**C**) α-eleostearic acid, (**D**) sophoricoside, (**E**) genistein, (**F**) formononetin. (**G**) The chemical structures and metabolic processing of hesperidin and its derivatives in vivo. (**H**) Hesperidin, a bioflavonoid compound, is mostly found in citrus fruits, including oranges, grapefruits, tangerines, and lemons. In the colon, hesperidin is converted into hesperetin by the microflora, and then hesperetin can be absorbed into systematic circulation by colonocytes. In the liver, hesperetin can be conjugated with a glucuronide or sulfate, which results in two other major metabolites, namely hesperetin-7-O-sulfate (H7S) and hesperetin-7-O-β-D-glucuronide (H7G), respectively. The figure was created with BioRender.com.

Curcumin (Figure 2B) is a bioactive yellow polyphenolic compound that is present in Curcuma longa rhizomes, which are harvested to produce the spice turmeric. Turmeric is widely applied in traditional Ayurvedic medicine and cooking. Several studies have characterized the beneficial effects of curcumin on natural aging and age-associated diseases, including diabetes, cancer, and neurodegenerative diseases. There are many beneficial effects of curcumin, including cardio-protection, nephron-protection, hepato-protection, anti-cancer effects, and neuroprotective properties; these are believed to be associated with the potent antioxidant and anti-inflammatory properties of this compound [68,69,70]. Intriguingly, previous studies indicated that curcumin treatment resulted in increased CISD2 expression in the SH-SY5Y cell line, which is a rat primary astrocyte cell line, and in the spinal cords of aged mice [38,71]. Lin et al. found that CISD2 expression was downregulated in the spinal cords of mice at 24 h after acute spinal cord injury (SCI).Curcumin treatment in the SCI-treated mice resulted in a decrease in inflammation-associated gene expression, namely expression of iNOS and RANTES, and was accompanied by an increase in CISD2 mRNA expression within the spinal cord [71]. Furthermore, curcumin treatment was found to increase the CISD2 protein level in the spinal cords of old mice. Moreover, it seems that curcumin-enhanced CISD2 expression is associated with the JAK/STAT signaling pathway of primary rate astrocytes [38]. These findings suggest that the neuroprotective effect of curcumin, in relation to spinal cord damage in mice, is associated with an enhanced expression of CISD2.

#### 4.3.2. WBM Extract, α-Eleostearic Acid, and ESM Powder

α-eleostearic acid (Figure 2C) is a type of linolenic acid that can be isolated from WBM. The chemical exhibits antioxidative, anti-inflammatory, and neuroprotective properties [72]. Using the SCI mouse model, Kung et al. found that WBM extract appears to reduce inflammation and rescue SCI-induced CISD2 downregulation in the spinal cord of mice. In addition, α-eleostearic acid had an anti-inflammatory effect that was accompanied by an increase in CISD2 mRNA expression using lipopolysaccharide-stimulated ALT astrocyte cell lines [73]. Moreover, using cDNA microarray analysis, Jia et al. found that dietary treatment with ESM powder or hydrated ESM for 14 days could bring about an improvement in liver fibrosis and increase CISD2 mRNA expression levels in the liver of young adult rats [74]. These studies suggest that WBM extract has an anti-inflammatory effect on the spinal cord, as well as an anti-fibrosis effect on the liver, and that these are likely to be associated with an increased expression of CISD2.

**Table 1:** Regimens or treatments that enhance CISD2 gene expression.

Compound or Intervention	Treatment and Duration	Tissue or Cell Line	Animal Model or Human Subjects	Enhance CISD2 Expression	Reference
**A. In Vitro Cell Study**					
Hesperetin	10 and 30 μM for 24 h	HEK293-CISD2 reporter cells		CISD2 reporter	[75]
Hesperetin -7-O-sulfate	30 μM for 24 h	HEK293-CISD2 reporter cells		CISD2 reporter	[75]
Curcumin	1 μM for 24 h	SH-SY5Y Rat primary astrocyte		CISD2 mRNA	[38]
Wild bitter melon extract	1 μg/mL for 24 h	Astrocyte cell line	LPS-challenged ACL	CISD2 mRNA	[73]
α-Eleostearic acid	0.28 μg/mL for 24 h	Astrocyte cell line	LPS-challenged ACL	CISD2 mRNA	[73]
Sophoricoside	10 and 30 μM for 24 h	HEK293-CISD2 reporter cells		CISD2 reporter	[75]
Genistein	10 and 30 μM for 24 h	HEK293-CISD2 reporter cells		CISD2 reporter	[75]
Formononetin	10 and 30 μM for 24 h	HEK293-CISD2 reporter cells		CISD2 reporter	[75]
**B. Animal Study **					
**Exercise**					
	Treadmill exercise for 8 weeks	Whole body (ventral view)	CISD2 reporter mice	CISD2 reporter	[66]
	Exercise with a running wheel for 4 weeks	Skeletal muscle eWAT	Male C57BL/6J mice	CISD2 protein	[67]
**Hesperetin**					
	100 mg/kg/day (provided in food) for 5 months	Heart Skeletal muscle	Aged mice (26 months old)	CISD2 protein	[75]
	100 mg/kg/day (provided in food) for 6 weeks	Whole body (ventral view)	CISD2 reporter mice	CISD2 reporter	[75]
**Curcumin**					
	40 mg/kg/day (i.p. injection) for 2 days	Spinal cord	Aged mice (24 months old)	CISD2 protein	[38]
	40 mg/kg/day (i.p. injection) for 2 days	Spinal cord	Spinal cord hemisectionin mice	CISD2 mRNA	[71]
**Miscellaneous**					
Wild bitter melon extract	500 mg/kg (i.p. injection) for single dose	Spinal cord	Spinal cord hemisection in mice	CISD2 mRNA and protein	[73]
ESM powder	Diet with 10 g/kg ESM powder for 14 days	Liver	Wistar rats	CISD2 mRNA	[74]
HEM powder	Diet with 10 g/kg HEM powder for 14 days	Liver	Wistar rats	CISD2 mRNA	[74]
**C. Human Study**					
RYGB surgery	3 months post-RYGB	Skeletal muscle	Obese females (BMI > 40 kg/m^2^)	CISD2 protein	[59]

## 5. Hesperetin Rejuvenates Aged Organs and Promotes Longevity

To translate our genetic evidence into a pharmaceutical application, it was important to systematically discover new regimens or compounds that could effectively increase CISD2 expression, and thus delay or even reverse the aging process [20]. Using HEK293-CISD2 reporter cells and CISD2 reporter mice, we identified several potential CISD2 activators that enhanced CISD2 gene expression at the transcriptional level, including sophoricoside (Figure 2D), genistein (Figure 2E), formononetin (Figure 2F), and hesperetin (Table 1 (A)). Among these compounds, hesperetin (a single compound with >98% purity) was identified as a promising CISD2 activator that enhanced CISD2 expression both in vitro and in vivo [75].

The 7-O-glycoside of hesperitin, namely hesperidin (Figure 2A), is the major flavanone-glycoside form of hesperetin and is found in various natural foods. Specifically, hesperidin and hesperetin are both well-known bioflavonoid compounds that are known to be present in numerous types of citrus fruits, including oranges (*Citrus sinensis*), grapefruits (*Citrus paradise*), tangerines (*Citrus reticulata*), limes (*Citrus aurantifolia*), and lemons (*Citrus limon*). Moreover, hesperidin is well-known to have a number of identified biological properties, including antioxidant, anti-bacterial, anti-inflammatory, and anti-carcinogenic effects [76]. However, previous studies indicated that the bioavailability of hesperidin is poor, due to its low water solubility and its limited absorption by the colon and intestines [77]. After oral intake of hesperidin, it is able to be hydrolyzed and de-glycosylated by the gut microflora into its aglycone form hesperetin; this mainly occurs in the colon. Once this has occurred, the hesperetin is absorbed into systematic circulation by colonocytes via proton-coupled active transport and transcellular passive diffusion [77]. In addition, plasma hesperetin is further metabolized into other metabolites, including hesperetin glucuronide (H7G, hesperetin-7-O-β-D-glucuronide) and hesperetin sulfate (H7S, hesperetin-7-O-sulfate) in the liver (Figure 2G,H). Our recent studies showed that hesperetin and its metabolite H7S are both able to enhance CISD2 expression [75].

### 5.1. Hesperetin Enhances CISD2 Expression and Promotes Longevity in Naturally Aged Mice

**Figure 3:**
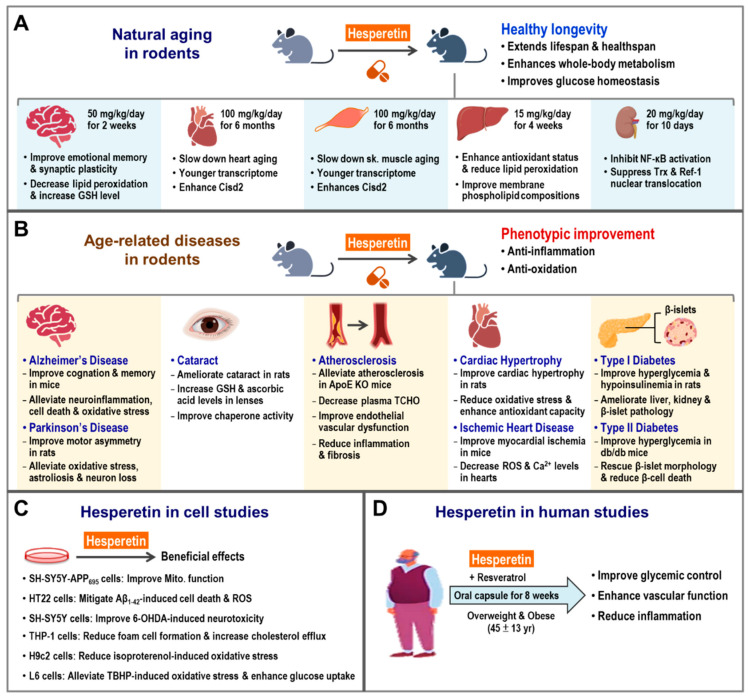
Beneficial effects of hesperetin on natural aging and age-related diseases in rodents and humans. (**A**) Hesperetin delays aging and promotes healthy longevity in rodents. There are many beneficial effects of hesperetin on the aging process in old mice, including improvements in healthy lifespan, whole-body metabolism, and glucose homeostasis. The beneficial effects of hesperetin on multiple tissues in aged rodents have been investigated and these include the brain, heart, skeletal muscle, liver, and kidneys. In the brain, hesperetin improves emotional memory functioning, hippocampal synaptic plasticity, and reduces oxidative stress in aged rats. In the hearts of older mice, hesperetin slows down heart aging, including an enhancement of mechanical and electrical cardiac functioning, reductions in fibrosis and ultrastructural damage, and change toward a younger transcriptome pattern. In the skeletal muscle of older mice, hesperetin slows down muscle aging, including improvements in muscle functioning; reductions in fibrosis, and levels of muscle fiber degeneration, and ultrastructural damage; and a change toward a younger transcriptome pattern. In the liver of aged rats, hesperetin reduces oxidative stress, enhances antioxidant enzyme activity, and improves the phospholipid composition of cell membranes. In the kidney of aged rats, hesperetin exerts anti-inflammation and antioxidant effects via the modulation of nuclear factor-κB (NF-κB) pathway. (**B**) Hesperetin exerts a range of biological effects that improve several age-related diseases in rodents, such as neurodegenerative diseases (Alzheimer’s and Parkinson’s disease), eye disease (cataracts), cardiovascular disease (atherosclerosis, hypertrophic cardiomyopathy, and ischemic heart disease), and metabolic syndrome, specifically diabetes. These phenotypic improvements are associated with the anti-inflammatory and antioxidant efficacies of hesperetin. (**C**) The mechanisms underlying the beneficial effects of hesperetin regarding age-related diseases in rodents were deciphered using different cell lines, such as neuronal cells (SH-SY5Y and HT22), immune cells (THP-1), cardiac myoblast cells (H9c2), β-cells (e.g., INS-1), and myoblast cells (L6). (**D**) In clinical studies, the efficacy and safety of hesperetin had been evaluated in overweight and obese human subjects. The biological efficacy of hesperetin in human subjects includes improved glucose homeostasis and vascular function and reduced inflammation. The figure was created with BioRender.com.

Remarkably, late-life treatment with dietary hesperetin, starting at 19–21 month old, has shown to enhance CISD2 gene expression and extend the lifespan and healthspan of mice. In addition, hesperetin treatment appears to attenuate whole-body metabolic decline, reducing fat and improving glucose homeostasis, as well as slowing down heart and skeletal muscle aging. RNA sequencing of heart and skeletal muscle revealed that hesperetin appeared to delay aging and bring about a younger pattern of gene expression in old mice. In CISD2 mcKO mice (MCK-Cre;CISD2 fl/fl), which carries a tissue-specific CISD2 KO affecting their skeletal and cardiac muscles, we found that the biological effects of hesperetin on age-associated phenotypes were mainly dependent on molecular mechanisms and pathways involving CISD2. Indeed, hesperetin lost its beneficial anti-aging effect when CISD2 could not be expressed in the skeletal muscle and heart of CISD2 mcKO mice [75]. In addition, a previous study from another group indicated that administration of hesperetin could reverse age-associated skeletal muscle degeneration and atrophy, as well as improve skeletal muscle performance and the redox-related GSH/GSSG ratio in old mice [2]. Together, these results suggest that hesperetin is a promising compound for the promotion of healthy longevity and the slowing down of aging via an enhancement of CISD2 expression in naturally aged mice (Figure 3A).

### 5.2. Hesperetin Slows Down Aging at the Organ Level

Hesperetin, an aglycone of hesperidin, is a type of flavonoid, which is abundantly present in the peels of citrus fruits. Numerous studies have shown that hesperetin exhibits potent antioxidant and anti-inflammatory properties [78,79,80,81]. The beneficial effects of hesperetin at the organ level have been reported in several studies of rodents (Figure 3A).

#### 5.2.1. Hesperetin and Brain Aging

Aging of the brain is associated with many structural alterations and functional decline. Previous studies have revealed that the integration of adaptive mechanisms (such as lifestyle and therapeutic interventions) is able to enhance brain health and prevent functional decline during old age. Such integrated interventions appear to promote stress resistance, increase synaptic plasticity, and maintain the functional integrity of the aging brain [82]. An animal study indicated that hesperetin seemed to improve age-associated brain defects, including emotional memory decline, hippocampal long-term potential changes, and synaptic plasticity impairments in aged rats. Moreover, hesperetin appears to reduce oxidative stress and increase glutathione levels in hippocampal tissue. These findings suggest that hesperetin can delay brain aging by maintaining redox homeostasis in old rats [83].

#### 5.2.2. Hesperetin and Liver Aging

The liver plays a pivotal role in mammalian aging. There are many age-associated phenotypes present in the aging liver, including various impairments of liver function, such as xenobiotic and lipid metabolism; the appearance of pathological changes such as hepatic steatosis, cell death, inflammation, and fibrosis; reduced regenerative capacity; and increased oxidative stress [84]. These are all easily detectable in the aging liver. In addition, aging predisposes mammals to several chronic or malignant liver diseases, including NAFLD, NASH, liver fibrosis, and liver cirrhosis, as well as liver cancer [85]. Therefore, slowing down liver aging would be able to ameliorate these age-associated liver disorders and prevent their progression. A previous study on aged rats revealed that hesperetin helped to maintain redox homeostasis, improve membrane phospholipid compositions, and enhance the activities of antioxidant enzymes in the liver [86]. Notably, hesperetin decreases various liver damage markers in old rodents, such as alanine aminotransferase (ALT) and aspartate aminotransferase (AST) [75,86]. Moreover, it seems that hesperetin has a beneficial effect where it helps to protect the liver against damage, attenuating inflammation and fibrosis [87]. These studies together support the hypothesis that hesperetin has an anti-aging effect on the liver.

#### 5.2.3. Hesperetin and Kidney Aging

Kidney aging is characterized by progressive structural and functional alterations; this age-associated damage accelerates the progression of chronic kidney disease and end-stage renal disease. Oxidative stress and inflammation both play important roles in kidney aging [88,89]. Based on the potent antioxidant and anti-inflammatory abilities of hesperetin, Kim et al. studied whether hesperetin was able to modulate NF-κB activation, which is associated with age-associated inflammation and oxidative stress in the kidneys of old rats. They found that hesperetin treatment led to an inhibition of NF-κB activation via its upstream regulators, namely the NIK/IKK, ERK, p38, and JNK signaling pathways. In addition, hesperetin suppressed the age-associated nuclear translocation of Trx and Ref-1, which are redox regulators of NF-κB. This suggests that hesperetin modulates the activities of the transcription factor NF-κB in a redox-dependent manner [90]. This study highlights hesperetin as a potential anti-aging compound for the kidney and age-associated kidney diseases.

### 5.3. Beneficial Effects of Hesperetin on Age-Associated Diseases

Based on the potent antioxidant, anti-inflammatory, and cell-protective properties of hesperetin, there have been many investigations of the therapeutic effects of hesperetin on age-associated diseases, including neurodegenerative, eye, cardiovascular, and metabolic diseases (Figure 3B). These have shown that hesperetin seems to be involved in the regulation of several aging-related signaling pathways, specifically the Nrf2/OH-1, Nrf2/ARE, TLR4/NF-κB, LXRα, AMPK, and Akt signaling pathways (Figure 3C).

#### 5.3.1. Hesperetin Improves Age-Associated Neurodegenerative Diseases

Aging is a major risk factor for neurodegenerative diseases, including AD and Parkinson’s disease (PD). Oxidative stress and neuro-inflammation are highly correlated with susceptibility to these neurodegenerative diseases. As hesperetin is a potent antioxidant and anti-inflammatory compound, several studies have evaluated the therapeutic effects of hesperetin on age-associated neurological disorders [79,91,92,93].

#### 5.3.2. Hesperetin and AD

Using an intra-cerebroventricular injection and the Aβ_1–42_-induced AD mouse model, Ikram et al. evaluated the effects of hesperetin on AD pathogenesis and characterized the underlying mechanisms. They found that hesperetin ameliorated Aβ_1–42_-induced cognitive and memory decline, as well as alleviated the pathological changes in the hippocampus and cortex, namely neuroinflammation, cell death, and oxidative stress (Figure 3B). Using the HT22 hippocampal cell line, hesperetin was found to decrease ROS levels and mitigate Aβ_1–42_-induced neurotoxicity. Mechanistically, the neuroprotective effect of hesperetin is likely to be mediated by enhancing Nrf2/OH-1 signaling and suppressing TLR4/NF-κB signaling [94]. Furthermore, using an in vitro SH-SY5Y-APP_695_ cell line model, Babylon et al. found that hesperetin reduced ROS levels and improved mitochondrial functioning, including increased oxygen consumption rate and ATP production [95] (Figure 3C). These findings indicate that hesperetin has neuroprotective properties, including the ability to improve neuroinflammation, reduce oxidative stress, and enhance mitochondrial function, all of which should help to limit AD pathogenesis.

#### 5.3.3. Hesperetin and PD

Using the intra-striatal 6-hydroxydopamine (6-OHDA)-induced PD model, Kiasalari et al. evaluated the protective effects of hesperetin on PD pathogenesis and explored the underlying mechanism. They found that hesperetin in rats could improve motor asymmetry and alleviate PD-related striatal damage, namely oxidative stress, neuroinflammation (astrogliosis), and hydroxylase-positive neuron loss [96] (Figure 3B). Furthermore, using the SH-SY5Y cell line model, Li et al. studied the underlying mechanism of the neuroprotective effects of hesperetin against 6-OHDA-induced neurotoxicity. They found that hesperetin seemed to ameliorate 6-OHDA-induced neurotoxicity, including cell death, oxidative stress, and GSH decline, via an enhancement of the NRF2/ARE signaling pathway [97] (Figure 3C). These studies support the hypothesis that hesperetin has neuroprotective abilities, including decreasing neuroinflammation and reducing oxidative stress, and that these effects are able to ameliorate PD pathogenesis.

#### 5.3.4. Hesperetin Protects against Age-Associated Eye Diseases

Cataracts are one of the main causes of age-associated blindness worldwide, which is characterized by lens degradation and clouding, as well as blurry and hazy vision [98]. The effects of hesperetin on the pathogenesis of cataracts have been evaluated. Using a selenite-induced cataract rat model, Nakazawa et al. studied the effects of hesperetin on the development of cataracts. They found that hesperetin ameliorates cataract pathogenesis in rats. In addition, hesperetin helped increase glutathione levels, restore redox status, improve chaperone activity, and maintain proteostasis (Figure 3B). However, the underlying protective mechanism by which hesperetin acts to prevent cataracts remains unclear [99,100,101].

#### 5.3.5. Hesperetin Improves Age-Associated Cardiovascular Diseases

Several studies have investigated the protective roles of polyphenols, including hesperetin, on heart failure [78,102]. The therapeutic effects of hesperetin on age-associated cardiovascular diseases are briefly described below.

In relation to atherosclerosis, using the apolipoprotein E knockout (ApoE KO) mouse model combined with a high-fat diet (HFD) treatment, Sugasawa et al. studied the beneficial effects of hesperetin on the development of atherosclerosis in mice. They found that hesperetin alleviated atherosclerotic progression in HFD-treated ApoE KO mice by decreasing serum total cholesterol levels, reducing inflammation of aortic roots, decreasing fibrosis affecting aortic roots, and controlling endothelial vascular dysfunction [103] (Figure 3B). Furthermore, hesperetin appeared to reduce foam cell formation and increase cholesterol efflux via an enhancement of the LXRα signaling pathway in the THP-1 macrophages [104] (Figure 3C). These findings indicate that hesperetin ameliorates atherosclerosis by suppressing inflammation and inhibiting macrophage-derived foam cell formation.

Cardiac hypertrophy and fibrosis are the two major features of cardiac aging [41]. When hesperetin and heart disease were investigated using the isoproterenol-induced cardiac hypertrophy rat model, Velusamy et al. were able to identify the beneficial effects of hesperetin with respect to cardiac dysfunction. They found that hesperetin ameliorated cardiac hypertrophy and fibrosis, reduced oxidative stress, and increased antioxidant capacity. These were characterized by increased antioxidant enzyme activity and an improved GSH/GSSG ratio in the rat heart (Figure 3B). Hesperetin mechanistically suppresses intracellular ROS production in the isoproterenol-treated H9c2 cardiac myoblast cell line via modulating the activation of the Nrf2/ARE signaling pathway [105] (Figure 3C). Furthermore, using the isoproterenol-induced myocardial ischemia mouse model, Liu et al. found that hesperetin had cardio-protective properties, namely that it improved myocardial ischemia and electrical functioning, and alleviated cardiac pathology, by mitigating oxidative stress and reducing inflammation. Moreover, the protective effect of hesperetin seemed to be associated with the activation of the Sirt1/Nrf2 pathway [106] (Figure 3B). These findings reveal that hesperetin has cardio-protective properties and is a potential therapeutic compound for mitigating age-associated cardiac diseases.

#### 5.3.6. Hesperetin Reduces Diabetes

Aging is a major risk factor for diabetes [107]. Previous studies have revealed that hesperetin seems to be a promising option for treating diabetes and diabetes-associated complications [79,80]. Using a streptozotocin (STZ)-induced diabetes rat model, Jayaraman et al. evaluated the beneficial effect of hesperetin on glucose homeostasis and diabetic-related pathology. They found that hesperetin alleviated hyperglycemia, reduced plasma insulin levels, and ameliorated STZ-induced pathology in the liver, kidneys, and β-islets [108]. Moreover, using the diabetic db/db mouse model, Wang et al. found that hesperetin improved hyperglycemia, protected pancreatic β-cells from cell death, and preserved β-islet morphology (Figure 3B). Using the INS-1 β-cell line, they found that hesperetin alleviated high glucose-induced β-cell death via activation of the AMPK pathway [109]. Furthermore, using the L6 myoblast cell line, Dhanya et al. found that the beneficial effects of hesperetin on diabetes were associated with enhanced glucose uptake and reduced oxidative stress via an upregulation of Akt expression [110] (Figure 3C). These findings show that hesperetin exhibits anti-diabetic properties, including maintaining glucose homeostasis, reducing oxidative stress, and helping to preserve β-cell functions; all of the above help to reduce the effects of diabetes.

#### 5.3.7. Hesperetin Reduces Age-Associated Metabolic Dysfunction

In humans, the effects of hesperetin on glucose homeostasis and vascular function in overweight or obese subjects was examined in a randomized, double-blind, placebo-controlled crossover clinical study (Healthy Aging Through Functional Food [HATFF]) (ClinicalTrials.gov Identifier: NCT02095873) [111,112]. Xue et al. evaluated the beneficial effects of a co-formulation, namely hesperetin (120 mg/day) plus trans-resveratrol (90 mg/day), on glycemic control and vascular function in a trial involving overweight (BMI > 27.5 kg/m^2^) and obese (BMI = 34.0 ± 0.7 kg/m^2^) subjects. The treatment involved consumption of an oral capsule once a day for 8 weeks. They found that, in the overweight and obese subjects, the co-formulated treatment of hesperetin and trans-resveratrol led to a reduced level of fasting plasma glucose, an increased oral glucose insulin sensitivity index, improvements in arterial vascular function, and decreased vascular inflammation markers [112]. These results show that oral administration of hesperetin and trans-resveratrol together for 8 weeks reduces certain age-associated metabolic dysfunctions in overweight and obese subjects, including dysglycemia, insulin resistance, and low-grade inflammation (Figure 3D).

## 6. The Anti-Aging Effect of Hesperetin Is Mainly Dependent on CISD2

The beneficial effects of hesperetin on aging and age-related phenotypes are largely dependent on CISD2, and hesperetin needs the presence of CISD2 to bring about its anti-aging effects [75]. Transcriptomic analysis by RNA sequencing of the skeletal muscle gastrocnemius revealed that most (79%) of the differentially expressed genes (DEGs) influenced by hesperetin were CISD2-dependent and these genes lost their differential expression patterns in the absence of CISD2. However, a smaller portion (21%) of the DEGs were CISD2-independent and these genes retained a differential expression pattern in the absence of CISD2 [75]. Overall, there were three major metabolic pathways in skeletal muscle that were affected by hesperetin; these included lipid metabolism, protein homeostasis, and nitrogen and amino acid metabolism (Table 2).

**Figure 4:**
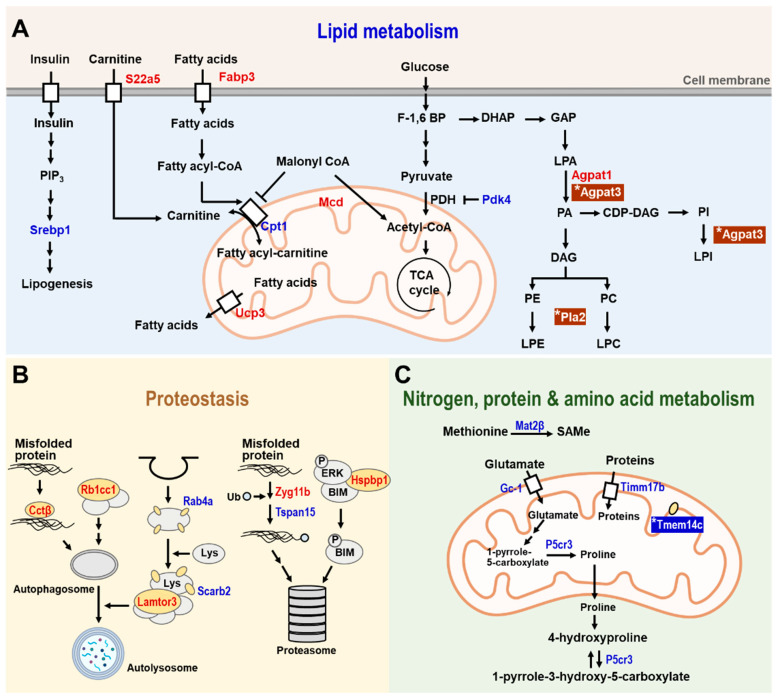
The CISD2-dependent and CISD2-independent metabolic pathways identified in the skeletal muscle of 3-month-old WT and CISD2 mcKO mice who undergo hesperetin treatment for 4 months. A schematic illustration of metabolism in the hesperetin-treated and vehicle-treated CISD2 mcKO mice that carry a CISD2 knockout background specifically affecting the skeletal and cardiac muscles. In aged skeletal muscle (gastrocnemius), hesperetin brings about improvements in various dysregulated pathways, namely (**A**) lipid metabolism, (**B**) proteostasis and (**C**) nitrogen, protein, and amino acid metabolism. Red indicates upregulation and blue indicates downregulation of the identified proteins. The presence of (*) emphasizes that these proteins were affected by hesperetin in a CISD2-independent manner. The figure was created with BioRender.com.

In lipid metabolism (Figure 4A), several genes that are involved in lipogenesis and fatty acid metabolism are regulated by hesperetin in a CISD2-dependent manner. These are listed below. First, sterol regulatory element-binding protein-1 (Srebp1) is an important transcription factor that is involved in inducing lipogenesis [113]. Second, fatty acid binding protein-3 (Fabp3), which is highly expressed in the heart and skeletal muscle, is involved in the uptake of fatty acids [114]. Third, uncoupling protein-3 (Ucp-3) is involved in the export of fatty acids from the mitochondrial matrix to cytosol [115]. Finally, both Fabp3 and Ucp-3 collaborate with carnitine palmitoyltransferase I (Cpt1), a key enzyme of mitochondrial beta-oxidation, to mediate fatty acid oxidation. Expression of all these genes in the skeletal muscle was affected by hesperetin treatment, and such treatment led to a remodeling of lipid metabolism and utilization in the skeletal muscle of WT mice, but not in the CISD2 mcKO mice.

On the other hand, only two genes have been identified after hesperetin treatment that are regulated in a CISD2-independent manner in lipid metabolism, namely 1-acylglycerol-3-phosphate O-acyltransferase 3 (Agpat3) and phospholipase A2, group XIIA (Pla2g12a). These two genes are involved in lysophosphatidic acid (LPA), phosphatidylethanolamine (PE), and phosphatidylcholine (PC) metabolism in skeletal muscle. These compounds are glycerophospholipids and are mainly involved in the maintenance of cellular membranes. They also play a role as signaling molecules under certain circumstances [116]. We found that Agpat3 and Pla2g12a were upregulated after hesperetin treatment in both WT and CISD2 mcKO mice, confirming that the effect of hesperetin on these two genes was independent of CISD2.

When proteostasis pathways were explored (Figure 4B), several genes involved in the autophagy and proteasomal degradation pathways were found to be regulated in a CISD2-dependent manner. These consisted of the following. First, chaperonin-containing TCP-1 subunit 2 (Cct2) facilitated the clearance of misfolded proteins by autophagy [117]. Second, retinoblastoma 1 inducible coiled-coil 1 (Rb1cc1), late endosomal/lysosomal Adaptor, MAPK, and mTOR Activator 3 (Lamtor3) all contributed to the formation of autophagosomes and autolysosomes [118,119]. Finally, Zyg-11 family member B (Zyg11b) and tetraspanin 15 (Tspan15) contributed to ubiquitin ligase activity [120,121]. Our findings revealed that expression of these age-related DEGs were reverted in the skeletal muscle of WT mice but not in the skeletal muscle of CISD2 mcKO mice after hesperetin treatment, which shows that hesperetin-mediated protein homeostasis in the skeletal muscle is mainly dependent on CISD2.

When nitrogen and amino acid metabolism were examined (Figure 4C), several genes involved in this area of metabolism were found to be regulated by hesperetin in a CISD2-dependent manner. First, translocase of the inner mitochondrial membrane 17b (Timm17b), which facilitates the transport of proteins from cytosol into the mitochondrial matrix, is regulated by CISD2. Additionally, there are other two important genes that are involved in glutamate metabolism, namely solute carrier family 25 member 22 (Slc25a22) and pyrroline-5-carboxylate reductase (Pycrl), which are also regulated in a CISD2-dependent manner. Another is Slc25a22, which is a mitochondrial glutamate transporter. Finally, Pycrl is an enzyme that catalyzes the conversion of 1-pyrrole-5-carboxylate to proline in the skeletal muscle. Glutamate is an anaplerotic precursor for the TCA cycle [122], and therefore CISD2-mediated glutamate metabolism is also involved in the oxidative energy production. In contrast to the above CISD2-dependent genes, transmembrane protein 14C (Tmem14c), which is an inner mitochondrial membrane protein [123], was regulated by a CISD2-independent pathway. Taken together, the above transcriptomic analyses support the hypothesis that the anti-aging effects of hesperetin are mainly dependent on CISD2.

## 7. Conclusions and Perspectives

As human life expectancy continues to increase around the world, aging and age-associated diseases are becoming a major global burden. The population of the world that is 65 years of age or older is projected to have tripled from the current 617 million (8.5% of the world population) to 1.6 billion (17% of the world population) by 2050. Based on the experimental results outlined above, it is clear that CISD2 plays an important role in the aging process, and a higher level of CISD2 appears to prevent age-associated organ dysfunction, prolong a healthy lifespan, and improve the quality of life during old age [124,125]. The functioning of CISD2 is related to many hallmarks of aging, both directly and indirectly. Accordingly, enhancement of CISD2 levels, whose expression otherwise decreases during natural aging, will have therapeutic benefits to individuals by attenuating and/or reversing age-related functional decline and structural damage, as well as ameliorating age-associated diseases.

The rates of age-dependent decrease of CISD2 vary across different tissues. Notably, at middle age (12 mo) in mice, CISD2 protein is downregulated in skeletal muscles. There are, on average, a 38 and 69% decrease in CISD2 protein in the femoris and gastrocnemius muscles, respectively [14,17,20]. At an older age (24 mo) in mice, the average decreases of CISD2 protein in the brain, skin, heart, and gastrocnemius muscle are 38, 40, 50, and 84%, respectively. However, the decrease in CISD2 protein in the kidney at 26 mo has been found to be less than 25%, which is lower compared with the other organs [20]. Growing evidence from studies on cross-talk between the skeletal muscles and other organs suggests that dysfunction of skeletal muscle appears to accelerate the progression of age-related diseases; this deteriorates the important functions that multiple organs have during aging [126,127]. Accordingly, the prevention of degeneration of skeletal muscle seems to be a good strategy when trying to attenuate aging because of the importance of inter-organ communication between skeletal muscle and other organs. Our findings reveal that CISD2 is a promising target for the development of therapeutic agents that, by bringing about an effective enhancement of CISD2 expression, will slow down aging. Moreover, enhancing CISD2 expression via treatment with CISD2 activators that attenuate the rapid decline of CISD2 in the skeletal muscle could be started as early as middle age so that normal inter-organ communication can be maintained during natural aging.

As a proof-of-concept, by showing that CISD2 can be pharmaceutically useful at a late-life stage, namely 19–21 months old in naturally aged mice, we provided evidence that hesperetin was a promising CISD2 activator that could slow down aging and promote longevity. The design and development of specific activators of CISD2 would be very practical for future studies and could also be commercially exploited for the treatment of age-associated diseases after necessary regulation. Last but not least, it is of great interest to know if there are any therapeutic alternatives to hesperetin that can also prevent the decrease of CISD2 gene expression during old age, so that CISD2 can be maintained at a high enough level to slow down aging.

## Figures and Tables

**Table 2:** DEGs influenced by hesperetin through CISD2-dependent or CISD2-independent pathways.

	CISD2 Dependent	CISD2 Independent
**A. Lipid Metabolism**		
Lipogenesis	Srebp1	
Fatty acid oxidation	Slc22a5, Fabp3, Cpt1, and Ucp3	
Malonyl CoA metabolism	Mlycd	
Pyruvate metabolism	Pdk4	
Phospholipid synthesis	Agpat1	Agpat3 and Pla2g12a
Others	Abcd3, Scd2, Pdss2, Cot7, Insig1, Synj2, Nceh1, and Pigq	
**B. Proteostasis**		
Ubiquitin proteasome system	Zyg11b, Tspan15, Hspbp1	
Autophagy-lysosome system	Rb1cc1, Cct2, Rab4a, Scarb2, and Lamtor3	
Others	Serp1, Paip1, Gspt1, Rpl34, Eif3g, Lrpprc, Mrps9, Cct4, Sugt1, Cox17, Cd24a, Rap1gds1, Kank1, Spag9, Pdss2, Nceh1, Fiz1, Adck1, Cript, and Ccdc47	
**C. Nitrogen, Protein & Amino Acid Metabolism**		
Methionine	Mat2b	
Glutamate	Slc25a22	
Proline	Pycrl	
Protein transport	Timm17b	
Others	Irf2, Smndc1, Zhx2, Cct4, Srebf1, Csde1, Mrps9, Tbx15, Ddx1, Pura, Acot7, Rap1gds1, Rb1cc1, Fiz1, Gspt1, Gm20390, Cct2, Hnrpll, Hist2h2aa1, and Lrpprc	Cops2, Hfe2, Taf9b, Slu7, and Tmem14c
D. Miscellaneous (Aging and Mitochondrial Function)		
Aging	Serp1 and Coq7	
Mitochondrial function	Cox17, Slc22a5, Immt, Cd24a, Synj2, Coq7, Lrpprc, Cox4i1, and Ndufb9	
Immune response and inflammation	Ddx1, Pdk4, Fgfbp1, Slc22a5, Sugt1, and Cd24a	

## Abbreviations

6-OHDA, 6-hydroxydopamine; ACL, astrocyte cell line; BMI, Body Mass Index; ESM, egg shell membrane; eWAT, epididymal white adipose tissue; LPS, lipopolysaccharide; GSH, reduced glutathione; HEM, hydrated ESM; NcPA, sodium cyclic lysophosphatidic acid; RYGB, Roux-en-Y gastric bypass; ROS, reactive oxygen species; TBHP, tertiary butyl hydrogen peroxide; TCHO, total cholesterol; wk, weeks; yr, years; AD, Alzheimer’s disease; ALT, alanine aminotransferase; Agpat1, 1-acylglycerol-3-phosphate O-acyltransferase 1; Agpat3, 1-acylglycerol-3-phosphate O-acyltransferase 3; AST, aspartate aminotransferase; BIM, BCL-2 interacting mediator of cell death; Cct2, chaperonin Containing TCP1 Subunit 2; CDP-DAG, cytidine diphosphate diacylglycerol; CISD2, CDGSH iron sulfur domain 2; Cpt1, carnitine palmitoyltransferase I; DAG, diacylglycerol; DHAP, dihydroxyacetone phosphate; eIF2α, eukaryotic translation initiation factor 2, subunit 1 alpha; ER, endoplasmic reticulum; F-1, 6 BP, fructose 1,6-bisphosphate; Fabp3, fatty acid binding protein 3; GAP, glyceraldehyde 3-phosphate; Gimap5, GTPase, IMAP Family Member 5; GO, gene ontology; HATFF, healthy aging through functional food; HCC, hepatocellular carcinoma; HFD, high fat diet; Hsbp1, heat shock protein beta-1; ICD, intercalated disc; IP3R, inositol 1,4,5-trisphosphate receptor; Lamtor3, late endosomal/lysosomal Adaptor, MAPK And MTOR Activator 3; LPA, lysophosphatidic acid; LPC, lysophosphatidylcholine; LPE, lysophosphatidylethanolamine; LPI, lysophosphatidylinositol; Lys, lysosome; MAM, mitochondrial-associated membrane; Mat2b, methionine adnosyltransferase 2B; Mlycd, malonyl-CoA decarboxylase; MOM, mitochondrial outer membrane; NAFLD, nonalcoholic fatty liver disease; NASH, non-alcoholic steatohepatitis; PA, phosphatidic acid; PC, phosphatidylcholine; PD, Parkinson’s disease; Pdk4, pyruvate dehydrogenase kinase 4; PE, phosphatidylethanolamine; PI, phosphatidylinositol; PIP3, phosphatidylinositol-3, 4, 5-triphosphate; Pla2g12a, phospholipase A2, group XIIA; Pycrl, pyrroline-5-carboxylate reductase; RAB4A, Ras-related protein Rab-4A; Rb1cc1, retinoblastoma coiled coil protein 1; SAMe, s-adenosyl-L-methionine; SCI, spinal cord injury; SR, sarcoplasmic reticulum; Scarb2, scavenger receptor class B member 2; Slc22a5, solute carrier family 22 member 5; Slc25a22, solute carrier family 25 member 22; Srebf1, sterol regulatory element binding transcription factor 1; Tmem14c, transmembrane Protein 14C; Timm17b, translocase of inner mitochondrial membrane 17B; Tspan15, tetraspanin 15; Ub, ubiquitin; Ucp3, uncoupling Protein 3; UPR, unfolded protein response; WD, western diet; WBM, wild bitter melon; WFS2, Wolfram syndrome 2; Zyg11b, zyg-11 family member B; H7G, hesperetin-7-O-β-D-glucuronide; H7S, hesperetin-7-O-sulfate; TM, transmembrane.
